# Age-dependent patterns of cardiac complexity unveiled by topological data analysis of pediatric heart rate variability

**DOI:** 10.1371/journal.pone.0337620

**Published:** 2025-12-02

**Authors:** Andy Domínguez-Monterroza, Alfonso Mateos Caballero, Antonio Jiménez-Martín

**Affiliations:** Department of Artificial Intelligence, Universidad Politécnica de Madrid, Madrid, Spain; University of Minnesota, UNITED STATES OF AMERICA

## Abstract

Heart rate variability (HRV) is a well-established marker of autonomic regulation and undergoes profound maturation during early human development. In this study, topological data analysis (TDA) is applied to investigate the evolving geometric complexity of HRV across pediatric developmental stages. Using persistent homology in homological dimension 1, we extracted topological descriptors from time-delay embedded RR interval series of 127 individuals aged 1 month to 17 years. We identified statistically significant, age-dependent transformations in the topological structure of HRV signals. Neonates and infants exhibited a greater number and strength of persistent features, reflecting highly heterogeneous cardiac control dynamics during early autonomic maturation. In contrast, adolescents displayed reduced topological complexity and increased entropy, suggesting a shift toward more uniform and structured physiological control. Topological measures correlated with conventional HRV indices, confirming their physiological relevance. Furthermore, pairwise distances between persistence landscapes revealed an inverse relationship between intra-group topological variability and classical HRV measures. Collectively, our findings demonstrate that persistent homology provides a powerful, multiscale-aware framework to capture developmental trajectories in cardiac autonomic regulation, with potential applications in pediatric monitoring, developmental physiology, and early detection of dysautonomia.

## 1 Introduction

Heart rate variability (HRV) is a well-established, non-invasive biomarker of autonomic nervous system (ANS) function, capturing the dynamic balance between sympathetic and parasympathetic modulation of cardiac activity [1,2]. In pediatric populations, HRV undergoes marked developmental transformations that parallel the progressive maturation of autonomic regulation from the neonatal period through adolescence [3–7]. Time-domain metrics, such as the *standard deviation of normal-to-normal* intervals (SDNN) and the *root mean square of successive differences* (rMSSD) typically increase during infancy, reach their peak in middle childhood, and stabilize or decline during adolescence, reflecting age-dependent reorganizations in ANS dynamics [5,8,9].

Despite clinical utility, conventional time and frequency-domain analyzes may fail to characterize the inherently complex, nonlinear, and multiscale nature of cardiac dynamics, particularly during early developmental windows marked by rapid physiological change [10]. To address these limitations, nonlinear methods such as *approximate entropy*, *sample entropy*, and *multiscale entropy* have been introduced to assess signal irregularity and complexity [11–13].

Complementary approaches, including fractal analyses - most notably *detrended fluctuation analysis* (DFA) - have been applied to quantify long-range temporal correlations and scale- invariant behavior in HRV time series [14]. However, these techniques generally rely on global statistical characterizations and may overlook fundamental geometric and structural properties embedded in the signal’s phase-space trajectory.

In addition, *Recurrence Quantification Analysis* (RQA) has been widely employed to characterize cardiac dynamics through recurrence patterns that reflect transitions between—and stability within—physiological states [15–18]. While RQA yields informative statistics (e.g., recurrence rate, determinism, laminarity), these primarily neighborhood-dependent, density-based measures provide only an indirect view of the global geometry of the reconstructed attractor. Consequently, RQA may be limited in detecting higher-order structural organization—such as cyclic connectivity and multiscale coordination—that can reveal global aspects of autonomic control. These considerations motivate the complementary use of TDA, which summarizes global structure across scales via persistent homology.

*Topological data analysis* (TDA) has recently emerged as a powerful paradigm to capture the underlying shape and structure of complex biological signals. Among TDA techniques, *persistent homology* provides a mathematically rigorous approach to identify and quantify topological features such as connected components and cycles across multiple spatial or temporal scales within embedded time series representations [19–22]. These descriptors have shown efficacy in diverse biomedical applications, including neural signal processing, cardiopulmonary monitoring, and speech dynamics [23,24].

Yet, the application of TDA to the study of HRV developmental trajectories remains largely unexplored. Although normative reference values of HRV across childhood and adolescence have been established using both linear and nonlinear techniques [5,6,9,14], the topological organization of HRV signals - and how it evolves through developmental stages - has not been systematically investigated. Given the sensitivity of persistence homology to features such as cyclicity, recurrence, and geometric transitions, TDA hold promise for revealing new dimensions of complexity in cardiac regulation during autonomic maturation.

The central hypothesis of this study is that topological descriptors derived from persistent homology can uncover age-dependent patterns of cardiac complexity that are not fully captured by conventional time-, frequency-, and nonlinear HRV indices. To test this hypothesis, we apply persistent homology to RR-interval time series from a cohort of healthy pediatric participants aged 1 month to 17 years, aiming to reveal developmental transitions in the geometry of cardiac dynamics. Our motivation is to provide multiscale, topology-based markers that complement standard HRV measures as objective indicators of autonomic maturation.Through delay-coordinate embedding, we reconstruct the dynamical state-space of each signal and compute topological descriptors in the first homology group (*H*_1_), which captures one-dimensional structures such as loops. These include the *number of persistent features*, *total persistence*, *mean persistence*, and *persistence entropy*. We then examine how these descriptors vary across defined developmental age groups to assess age-dependent changes in cardiac structure.

The remainder of this paper is organized as follows. Section 1 reviews the relevant literature on heart rate variability and topological data analysis. Section 2 describes the methodology, including the dataset, preprocessing steps, feature extraction with persistent homology, and analytical techniques. Section 3 presents the results of the topological analysis. Section 4 discusses the implications of these findings, their relation to existing research, and potential physiological interpretations. Finally, Section 5 summarizes the main conclusions, highlights the study’s contributions, and suggests directions for future research.

## 2 Literature review

The assessment of HRV in pediatric populations has progressively evolved from traditional linear methodologies toward more advanced analytical frameworks aimed at capturing the intricate dynamics of autonomic maturation. Throughout childhood and adolescence, HRV exhibits marked age-dependent modulations that reflect the structural and functional development of cardiac autonomic regulation systems [4,6,8].

Conventional time-domain (e.g., SDNN, RMSSD, pNN50) and frequency-domain (e.g., LF, HF, LF/HF ratio) indices have provided foundational insights into these developmental patterns [4–6,8,14]. However, their underlying assumptions of linearity and signal stationarity limit their interpretability in pediatric contexts characterized by inherently dynamic, nonlinear, and multiscale physiological changes [10].

In response to these limitations, nonlinear analytical frameworks have been increasingly employed to capture the complexity and adaptive properties of cardiac autonomic function. Seminal studies, such as that of Cysarz et al. [25] demonstrated significant nonlinear developmental trajectories using entropy measures and DFA on long-term HRV recordings, revealing non-monotonic trends in fractal scaling (DFA $\alpha_{1}$) peaking in mid-childhood. These findings challenged conventional linear interpretations, highlighting the intrinsic complexity of autonomic maturation.

Recent normative studies have further expanded the analytical landscape by integrating indices derived from 24-hour Holter recordings [26]. These have consistently shown age-related reductions in entropy and fractal self-similarity, reinforcing the need for multidimensional approaches that account for temporal, spectral, and nonlinear dimensions variability. Clinical studies have additionally validated the diagnostic utility of nonlinear HRV measures in developmental psychopathology. For instance, Fiskum et al. [9] reported that entropy-based indices effectively differentiated children with internalizing disorders from healthy controls, while other investigations underscored the value of fractal and recurrence analyses as sensitive markers of autonomic dysregulation in pediatric populations [27].

Comparative studies have emphasized the methodological robustness of combining multiple nonlinear measures — such as multifractal DFA, entropy metrics, and Poincaré plot analysis — over single-metric approaches, particularly in pediatric cohorts where developmental dynamics are highly heterogeneous [28]. Beyond classical nonlinear approaches, methodological advances have extended HRV analysis to symbolic dynamics, temporal asymmetry, and fragmentation frameworks. Symbolic dynamics —often via entropy-like symbolic indices—encodes ordinal patterns in beat-to-beat variability, capturing short-term organization and regime shifts within the cardiac signal [29]. Asymmetry metrics quantify directional differences between heart-rate accelerations and decelerations, providing markers of sympathetic–parasympathetic balance [30,31]. Heart-rate fragmentation characterizes abrupt, short-lived, and stochastic oscillations in RR intervals that may indicate disrupted or immature autonomic regulation [32]. These methods yield sensitive descriptors of local temporal structure and regulation. Complementarily, TDA summarizes the global, multiscale geometry of the reconstructed dynamics via persistent homology (e.g., *H*_0_, *H*_1_ features), offering noise-robust topological invariants that augment symbol-, metric-, and fragmentation-based measures.

Furthermore, sex- and age-specific analyses have revealed consistent gender differences in nonlinear HRV indices, as well as notable transitions in fractal complexity during pubertal onset [14,33], contributing to more precise age-appropriate reference models.

Beyond developmental physiology, nonlinear HRV measures have demonstrated relevance in capturing autonomic alterations associated with emotional and psychological states. Studies in young adults have shown that state anxiety is associated with detectable changes in HRV structure, particularly through correlation dimension, Lyapunov exponents, and entropy measures, supporting potential applications in pediatric stress and emotion monitoring [27]. Complementarily, Gasior et al. [5] emphasized the need to normalize HRV indices relative to heart rate given age-related variations in baseline rhythm, while other authors have highlighted the modulating effects of environmental and behavioral factors such as physical activity, sleep, and body composition [8].

Despite these advances, most nonlinear methods yield scalar summaries of signal complexity and fail to capture the geometric and topological structure embedded in the temporal organization of RR intervals. Topological data analysis — and in particular, persistent homology — has emerged as a powerful mathematical framework capable of quantifying multiscale topological features, offering scale-invariant and noise-robust descriptors of signal structure [34–37]. Although persistent homology has proven effective in differentiating pathological from healthy HRV patterns in adult populations (e.g., post-stroke cases [36]), its application to pediatric HRV analysis remains largely unexplored.

Notably, prior pediatric studies such as those by Lavanga et al. [6] and Harteveld et al. [4] have advanced our understanding of age-dependent HRV maturation using traditional linear and nonlinear metrics, but without addressing the topological and geometric evolution of cardiac autonomic regulation. This gap is critical, as persistent homology enables the detection of structural transitions, recurrence patterns, and hierarchical organization in signal dynamics, properties that are particularly relevant during the rapid physiological changes of early life.

Accordingly, the present study aims to bridge this methodological gap by integrating persistent homology into the analysis of HRV maturation. We apply TDA to RR interval time series from a cross-sectional pediatric cohort (1 month to 17 years of age), reconstructing the underlying dynamical systems via delay-coordinate embedding and extracting a set of topological descriptors in the first homology group (*H*_1_), including the number of persistent features, total, maximum and mean persistence, and persistence entropy. These descriptors are analyzed across developmental age groups and compared with standard HRV indices to evaluate their physiological relevance.

Our findings reveal significant age-related shifts in topological HRV complexity, with early stages exhibiting high topological heterogeneity and adolescents demonstrating more structured and compact signal architectures. Topological features also show meaningful correlations with conventional HRV indices, underscoring their validity as biomarkers of autonomic maturation. This integrative topological framework offers novel insights into pediatric autonomic maturation and holds promise for refining normative references and enhancing clinical assessment tools in pediatric physiology and developmental medicine.

## 3 Methods

**Fig 1:**
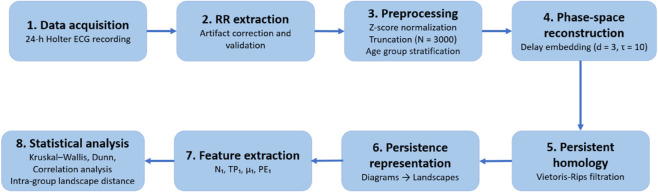
Overview of the analytical workflow.

This study employed a TDA framework to investigate developmental changes in HRV. The methodological workflow comprised six sequential stages, which are detailed in the following subsections. First, we curated a publicly available dataset of RR interval time series from a pediatric cohort [38,39]. The signals underwent preprocessing and quality control to ensure artifact-free data. Participants were then stratified into developmental age groups according to established pediatric definitions. Next, we extracted multiscale topological descriptors from time-delay embeddings of the HRV signals using persistent homology. Group-level differences were quantified through persistence landscapes to capture developmental trajectories. Finally, statistical analyses were performed to evaluate inter-group differences and to examine correlations between topological features and conventional HRV metrics. Fig 1 provides an overview of the analytical workflow, summarizing the six stages from data acquisition to topological feature extraction and statistical analysis.

### 3.1 HRV dataset and participants

Heart rate variability time series were obtained from a publicly available dataset hosted on PhysioNet [38,39]. For this study, we included only participants aged 1 month to 17 years to investigate age-dependent changes in cardiac autonomic function during early development, yielding a final sample of 127 individuals. Participants were excluded if age information was unavailable or if essential data were missing.

All data were collected with institutional ethics approval from the National University of La Plata, Argentina. Participants were medication-free and exhibited normal electrocardiographic profiles according to standard Holter monitoring guidelines.

### 3.2 Signal acquisition and preprocessing

Twenty-four-hour ECG recordings were acquired using digital three-lead Holter monitors (DMS300-7, DMS300-3A, Galix), with sampling frequencies ranging from 512 to 1024 Hz. RR intervals were extracted from sinus rhythm segments, and artifacts were identified and corrected by expert cardiologists. Only recordings with fewer than 8% total artifact burden and no individual artifact segment exceeding 20 seconds were included. Artifact-contaminated segments were excluded, and only clean, continuous RR interval time series were retained for analysis.

No additional digital filtering was applied beyond artifact correction, ensuring that analyses were conducted on physiologically valid, minimally processed data and that intrinsic beat-to-beat variability was preserved. For each participant, we selected a contiguous, artifact-free segment of 3,000 RR intervals. Because the segment length is defined in beats, its duration depends on the average heart rate and thus spans approximately 25–43 minutes across our cohort (≈ 3,000/120–3,000/70 min; infants ≈ 120 bpm, adolescents ≈ 70 bpm). This window provides sufficient data for reliable estimation of topological descriptors while standardizing data quantity across developmental stages. Prior to state-space reconstruction, each RR series was z-score standardized on a per-subject basis to remove baseline heart-rate differences, ensuring that extracted topological features reflect intrinsic temporal dynamics rather than absolute level.

### 3.3 Developmental age stratification

To evaluate age-related trends in HRV dynamics, participants were stratified into seven developmentally meaningful age groups (see Table 1): *neonates* (0–1 month), *early infancy* (1–5 months), *late infancy* (6–11 months), *toddlers* (1–2 years), *preschoolers* (3–5 years), *school-age children* (6–11 years), and *adolescents* (12–17 years). These strata were designed to capture key stages of the autonomic nervous system and cardiovascular development.

**Table 1:** Distribution of participants across defined developmental age groups.

Age group	Sample size (n)
Neonates (0–1 mo)	8
Early infancy (1–5 mo)	33
Late infancy (6–11 mo)	29
Toddlers (1–2 yr)	22
Preschoolers (3–5 yr)	10
School-age (6–11 yr)	15
Adolescents (12–17 yr)	10
**Total**	**127**

### 3.4 Topological feature extraction

To quantify the multiscale structural properties of heart rate variability signals, we employed TDA using persistent homology, a computational framework derived from algebraic topology. This method characterizes the shape of data by identifying persistent topological features that are robust to noise and provide a signature of the underlying dynamics [40].

Each RR interval time series $\left\{RR_{1},RR_{2},...,RR_{N}\right\}$ was transformed into a point cloud in a reconstructed phase space using the method of time-delay embedding via Takens’ embedding theorem [41]. This theorem guarantees that for a sufficiently high embedding dimension, the topological properties of the unknown original dynamical system that generated the time series are preserved in the reconstructed space. Each point ${𝐯}_{i}$ in this cloud is defined as:

$${𝐯}_{i}=(RR_{i},RR_{i+\tau },RR_{i+2\tau },...,RR_{i+(d-1)\tau }),$$ (1)

where *d* is the embedding dimension and *τ* is the time delay.

The choice of embedding parameters was guided by physiological considerations and established practices in nonlinear HRV analysis. The time delay (*τ*) was estimated using average mutual information [42], selecting the first minimum of statistical dependence between successive samples; across developmental groups, the median optimal delay ranged 7–12 beats, consistent with the first zero-crossing of the autocorrelation function reported for healthy HRV signals [43]. We therefore adopted *τ* = 10 beats, ensuring that successive coordinates (*RR*_*i*_,*RR*_*i* + 10_,... ) are sufficiently decorrelated while retaining short-term autonomic influences. This selection is also consistent with the geometry of lagged Poincaré plots, where the structure at lag m reflects the signal’s autocovariance at that lag [44]. In temporal terms, for typical pediatric heart rates (≈ 60–120 bpm), this corresponds to 5–10 s, an appropriate scale to capture respiratory sinus arrhythmia and baroreflex modulation.

The embedding dimension *d* was evaluated via False Nearest Neighbors (FNN) and Cao’s quantitative measures [45]. FNN fell below 5% at d≈6, and Cao’s ratios saturated near d ≈ 9, indicating adequate unfolding in a low-dimensional space. Prior studies show that topological/geometric invariants of time series often stabilize at smaller embedding dimensions, revealing essential dynamical structure without redundant coordinates [46]. Consistent with widespread practice in nonlinear HRV, we adopted a three-dimensional embedding (*d* ≈ 3) to obtain a compact, interpretable phase space and to facilitate extraction of topological features (e.g., *H*_1_ loops in 3D) linked to oscillatory components of autonomic regulation, while avoiding the combinatorial complexity and noise sensitivity of higher-dimensional reconstructions [47,48].

This evidence supports the final parameter combination (*d* = 3, *τ* = 10) as a physiologically meaningful and computationally efficient representation of reconstructed cardiac dynamics, yielding stable and interpretable topological summaries suitable for characterizing autonomic maturation. A complementary sensitivity analysis (S1 File) confirmed that the reconstructed attractor retains a stable topological organization at *d* = 3, with higher-dimensional embeddings contributing minimal additional information at increased computational cost.

From each embedded time series, *Vietoris–Rips filtrations* were applied to construct persistence diagrams in homological dimension 1 (*H*_1_). This homological dimension encodes one-dimensional topological features - specifically, loops and cycles - within the reconstructed state space. These features reflect the recurrent and oscillatory dynamics intrinsic to HRV, governed by feedback mechanisms of the autonomic nervous system. Focusing on *H*_1_ is particularly relevant in physiological time series analysis, as it captures temporally structured recurrence patterns that are not readily detectable through conventional linear or spectral methods. Prior evidence supports the relevance of homological dimension one in distinguishing physiologically distinct states [37].

From each persistence diagram *D*_*k*_, we extracted the following quantitative descriptors of topological complexity:

- *Number of Persistent Features* (*N*_*k*_). The total number of topological features (e.g., cycles in dimension *k* = 1) in the diagram. Reflects the structural richness or complexity of the signal geometry:
  $$N_{k}=|D_{k}|.$$
- *Total Persistence* (*TP*_*k*_). The cumulative lifespan of all persistent features, quantifying the overall prominence of the topological structures:
  $$TP_{k}=\sum_{(b_{i},d_{i})\in D_{k}}(d_{i}-b_{i}).$$ (2)
- *Maximum Persistence* (*MP*_*k*_). The single longest-lived feature, indicating the most dominant cyclic structure in the signal:
  $$MP_{k}=\max_{(b_{i},d_{i})\in D_{k}}(d_{i}-b_{i}).$$ (3)
- *Mean Persistence* ($\mu_{k}$). The average lifetime of features in the persistence diagram, representing typical cycle prominence:
  $$\mu_{k}=\frac{1}{N_{k}}\sum_{(b_{i},d_{i})\in D_{k}}(d_{i}-b_{i}).$$ (4)
- *Persistence Entropy* (*PE*_*k*_). A Shannon entropy-based metric quantifying the diversity or disorder of persistent features, normalized by their relative contribution to total persistence:
  $$PE_{k}=-\sum_{i=1}^{N_{k}}p_{i}\log(p_{i}),\;\text{where}\;p_{i}=\frac{d_{i}-b_{i}}{TP_{k}}.$$ (5)

These metrics jointly quantify abundance, stability, and diversity of cyclic structure in the reconstructed phase space, providing interpretable and complementary summaries of autonomic oscillations. All descriptors were computed on z-score standardized RR series using the same filtration and metric to ensure comparability across subjects and ages, following established practice and stability results for persistent homology.

All topological descriptors were computed using the *Ripser* library in Python [49], with customized post-processing routines to standardize output formats and remove spurious low-persistence features where necessary.

### 3.5 Quantifying topological dissimilarity between age groups via persistence landscapes

To evaluate inter- and intra-group differences in the topological structure of HRV dynamics across developmental stages, we employed persistence landscapes — a functional summary representation of persistence diagrams introduced by Bubenik [50].

A *persistence landscape* ℒ is defined as a sequence of functions $\{\lambda_{k}(t){\}}_{k=1}^{\infty }$, where each function $\lambda_{k}:\mathbb{R}\to {\mathbb{R}}_{\geq 0}$ denotes the *k*-th largest *tent function* evaluated at filtration scale *t*. This transformation encodes the multiscale topological features of a persistence diagram into a structured, Hilbert-space-compatible representation suitable for statistical comparison.

For computational tractability, each landscape was discretized by evaluating the first *k* = 1 landscape function over a fixed grid of *N* = 100 uniformly spaced filtration values. The result is a vectorized representation ℝ:

$$ℒ={\left[\lambda_{1}(t_{1}),\lambda_{1}(t_{2}),\ldots ,\lambda_{1}(t_{N})\right]}^{⊤}\in {\mathbb{R}}^{N},$$ (6)

where $\left\{t_{1},t_{2},\ldots ,t_{N}\right\}$ denotes the discrete filtration scale grid.

To quantify topological dissimilarity between pediatric age groups, we computed the average pairwise Euclidean distance between persistence landscapes derived from the first homology group of time-delay embedded HRV signals.

Let *G*_*i*_ and *G*_*j*_ denote two developmental age groups, and let ${ℒ}_{a}$ and ${ℒ}_{b}$ be the corresponding persistence landscapes of subjects $a\in G_{i}$ and $b\in G_{j}$, respectively. The mean pairwise topological distance between groups *G*_*i*_ and *G*_*j*_ is defined as:

$${\bar{d}}_{G_{i},\;G_{j}}=\frac{1}{\left|G_{i}||G_{j}\right|}\sum_{a\in G_{i}}\sum_{b\in G_{j}}d({ℒ}_{a},{ℒ}_{b}),$$ (7)

where d(·,·) denotes the Euclidean distance between the discretized landscapes vectors:

$$d_{Euc}({ℒ}_{a},{ℒ}_{b})={\left(\sum_{k=1}^{N}{\left({ℒ}_{a}^{\left(k\right)}-{ℒ}_{b}^{\left(k\right)}\right)}^{2}\right)}^{1/2}.$$ (8)

The procedure yields a symmetric distance matrix summarizing the pairwise topological dissimilarities between all developmental groups. Each matrix entry (*i*,*j*) represents the mean Euclidean distance between the persistence landscapes of individuals in groups *G*_*i*_ and *G*_*j*_, thereby quantifying the degree of divergence in the recurrent geometrical patterns of their HRV dynamics.

Diagonal elements (*i*, *i*) capture the average within-group topological variability, providing insight into how heterogeneous the cardiac signal structure is among individuals of the same developmental stage. Off-diagonal elements (*i*, *j*), on the other hand, reflect between-group differences: larger values indicate greater dissimilarity in topological organization across age groups. This framework enables a joint assessment of both intra-group coherence and inter-group divergence in the structural complexity of HRV signals.

### 3.6 Statistical analysis

To evaluate age-dependent differences in conventional HRV indices and topological descriptors derived from persistent homology, we performed non-parametric groupwise comparisons using the Kruskal–Wallis H test. When the overall test indicated statistically significant differences among age groups, post hoc pairwise comparisons were carried out using Dunn’s test with Holm–Bonferroni correction to control for multiple testing.

To examine the relationship between topological descriptors and conventional HRV indices, such as SDNN, RMSSD, pNN50, we computed Pearson correlation coefficients. Prior to correlation analysis, all indices were log-transformed to approximate normality and stabilize variance.

All statistical analyses were performed using Python (version 3.12), with relevant packages including: scipy, scikit-posthocs, and statsmodels. A two-tailed significance threshold of *p* < 0.05 was applied throughout.

## 4 Results

To evaluate the proposed approach, we analyzed the HRV recordings across the different pediatric age groups and derived their corresponding topological descriptors. The following subsections summarize the main outcomes of this analysis, including the age-dependent transformations observed in the persistence features, their relationship with conventional HRV indices, and the variability patterns across groups.

### 4.1 Conventional HRV indices across developmental stages

To contextualize the topological findings, we first evaluated conventional time-domain HRV metrics across age groups (Table 2). Kruskal–Wallis tests revealed significant between-group differences for Mean RR (*p* < 0.001), SDNN_*RR*_ (*p* < 0.001), and pNN50_*RR*_ (*p* < 0.001), whereas RMSSD_*RR*_ did not reach statistical significance (*p* = 0.115). Post-hoc Dunn comparisons showed a monotonic increase in Mean RR from 404 ms in neonates to 674 ms in adolescents, consistent with the well-known age-related slowing of heart rate. SDNN_*RR*_ and pNN50_*RR*_ were lowest in infant groups and highest in school-age children and adolescents, in line with established maturation patterns whereby overall HRV increases through childhood before stabilizing or slightly declining in adolescence. These findings confirm that our cohort captures fundamental developmental physiology and provide a benchmark against which to interpret the novel topological descriptors.

**Table 2:** Group-wise summary of conventional HRV indices (median [IQR]) across pediatric developmental stages.

Age Group	Mean RR (ms)	SDNN_*RR*_ (ms)	RMSSD_*RR*_ (ms)	PNN50_*RR*_ (%)
Neonates (0–1 mo)	404 [395–417]	62.9 [57–66]	67.9 [61–73]	4.2 [3.9–4.4]
Early Infancy (1–5 mo)	402 [395–412]	40.9 [38–44]	26.6 [25–30]	1.2 [0.9–1.4]
Late Infancy (6–11 mo)	431 [423–438]	36.7 [34–40]	32.4 [30–35]	1.4 [1.1–1.8]
Toddlers (1–2 yr)	474 [467–482]	49.4 [45–53]	52.7 [48–55]	2.9 [2.6–3.2]
Preschoolers (3–5 yr)	508 [500–515]	55.6 [51–59]	30.7 [28–33]	4.1 [3.8–4.3]
School-age (6–11 yr)	609 [602–624]	70.9 [68–75]	40.7 [38–43]	15.2 [14–17]
Adolescents (12–17 yr)	674 [669–680]	81.4 [78–85]	39.2 [37–41]	14.1 [13–15]
**Kruskal–Wallis *p***	<0.001	<0.001	0.11	<0.001

### 4.2 Topological complexity of HRV exhibits age-dependent patterns

**Fig 2:**
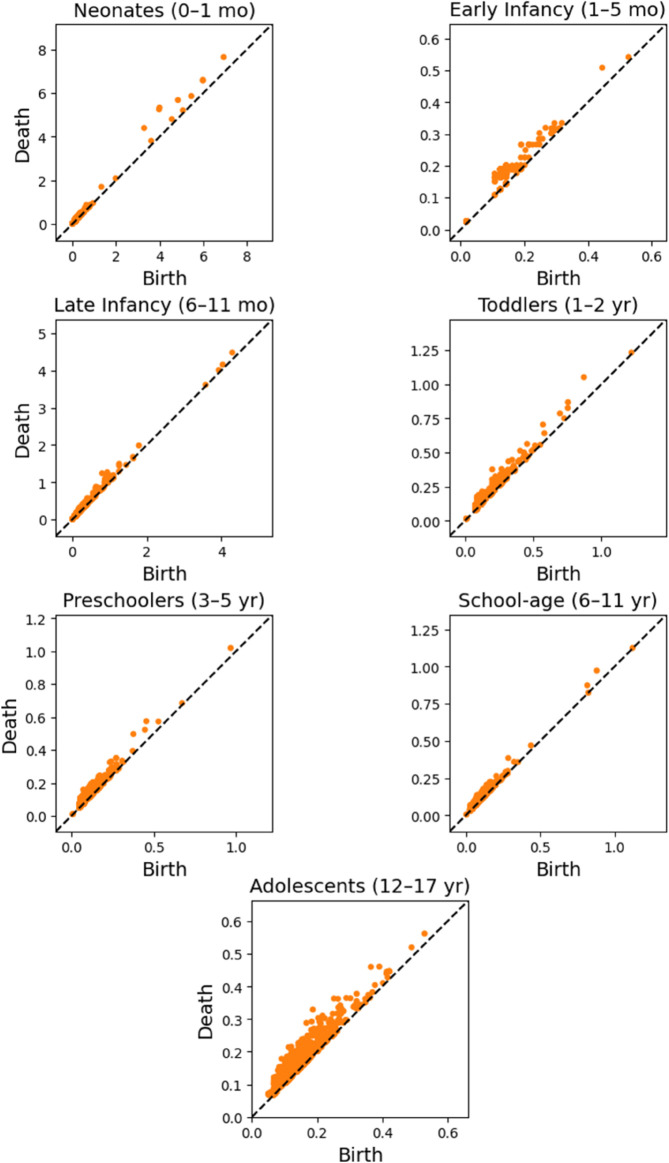
Representative persistence diagrams (*H*_1_) of time-delay embedded HRV signals across developmental age groups.

To investigate the structural evolution of cardiac autonomic regulation during development, we analyzed the one-dimensional topological features of HRV signals across pediatric age groups using persistent homology. Representative persistence diagrams for each developmental stage are presented in Fig 2, where each point (*b*,*d*) denotes the birth and death of homological cycle in the embedded HRV signal. The vertical distance from the diagonal (*d*–*b*) represents the persistence, or prominence, of the corresponding feature. Longer-lived features (further from the diagonal) reflect more persistent topological cycles.

**Fig 3:**
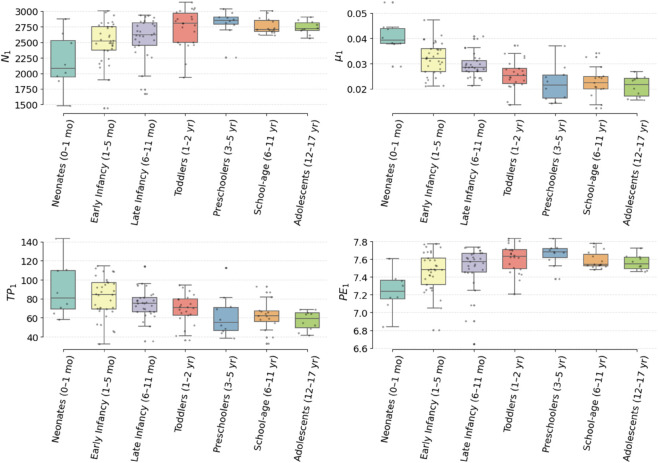
Boxplots of topological descriptors across pediatric age groups.

Notably, neonatal subjects exhibit fewer topological features, but with longer persistence, suggesting a smaller number of dominant, well-defined oscillatory patterns. In contrast, older children and adolescents show denser distributions of short-lived cycles, indicating a transition toward more fragmented and temporally constrained dynamics. This visual pattern suggests that the maturation of autonomic regulation may involve a topological shift from strong, recurrent structures to a larger number of less persistent cycles. To quantify these observations, we computed five topological descriptors from the persistence diagrams in homology dimension one: number of persistent features (*N*_1_), total persistence (*TP*_1_), maximum persistence (*MP*_1_), mean persistence ($\mu_{1}$), and persistence entropy (*PE*_1_). The distributions of these descriptors across age groups are shown in Fig 3, and the corresponding statistical results are summarized in Table 3 (For more information see supplementary information in S2 File). Neonates exhibit lower topological complexity and higher entropy in cycle structure compared to older groups.

**Table 3:** Kruskal–Wallis and Dunn’s post-hoc test results for topological descriptors.

TDA metric	*p*-value	Significant differences
*N*_1_	<0.001	Neonates vs. Preschoolers, Toddlers
*TP*_1_	<0.001	Adolescents vs. Early infancy; Neonates vs. School-age
*MP*_1_	0.0048	Neonates vs. Adolescents, School-age
$\mu_{1}$	<0.001	Neonates vs. all older groups except Toddlers
*PE*_1_	<0.001	Neonates vs. Preschoolers, Toddlers

Statistical analysis using the Kruskal–Wallis test revealed significant groupwise differences for all descriptors (*p* < 0.005 for all metrics). Post hoc Dunn’s comparisons further identified specific developmental contrasts, particularly between neonates and older groups. For instance, neonates showed significantly lower *N*_1_ and *PE*_1_, but higher *MP*_1_ and $\mu_{1}$, compared to toddlers and preschoolers, indicating fewer but more pronounced topological features with lower structural disorder. These results suggest that early HRV signals exhibit globally simpler but more coherent dynamic patterns, which become increasingly fragmented and variable with age.

Interestingly, while older age groups (school-age and adolescents) exhibited lower values of *TP*_1_ and flatter distributions of ℒ(t), they also displayed reduced interindividual variability, as reflected in narrower interquartile ranges in Fig 3. This supports the hypothesis that autonomic function becomes not only less complex but also more topologically homogeneous with age.

**Fig 4:**
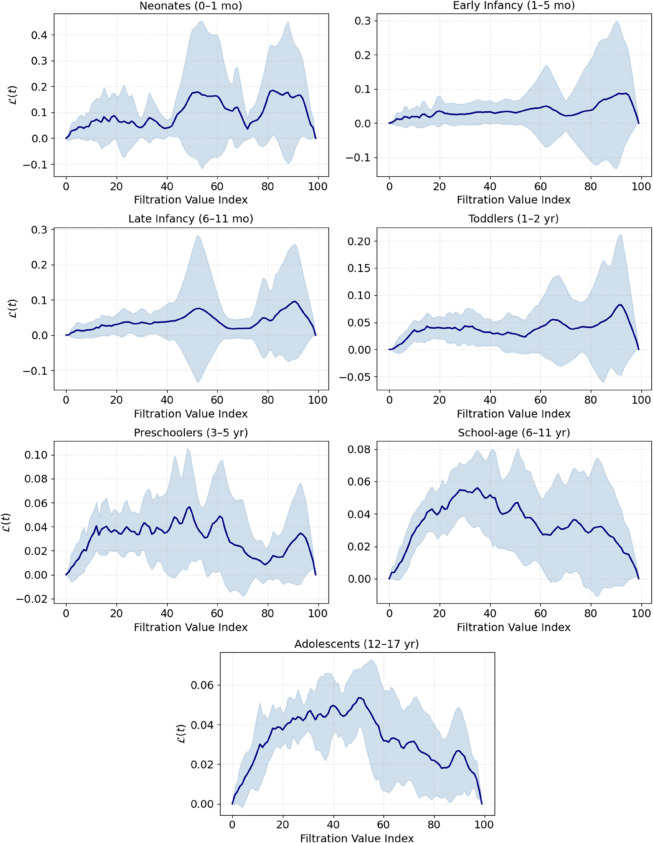
Group-averaged persistence landscapes ℒ(t) for dimension *H*_1_.

To further characterize group-level topological structure, we computed group-averaged persistence landscapes ℒ(t), shown in Fig 4. These curves summarize the average strength and recurrence of topological cycles across filtration scales for each age group. Each curve represents the mean landscape across individuals within an age group, with shaded areas indicating ±1 standard deviation. Lower topological amplitude and flatter profiles in older groups reflect reduced cycle strength and recurrence in cardiac dynamics.

The youngest groups (neonates, early infancy, and late infancy) showed markedly higher and more variable landscape amplitudes, indicating greater recurrence and diversity of loop-like features in their HRV geometry. Older groups, particularly school-age children and adolescents, exhibited reduced landscape heights and flatter profiles, reflecting more uniform cardiac dynamics and fewer dominant oscillatory patterns.

**Fig 5:**
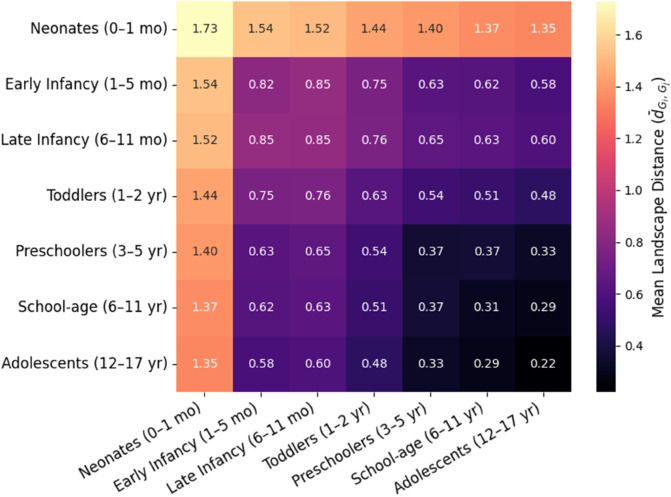
Matrix of pairwise Euclidean distances between persistence landscapes across age groups.

To assess topological dissimilarity between age groups, we computed pairwise Euclidean distances between individual persistence landscapes and averaged them within and between groups. The resulting symmetric distance matrix is shown in Fig 5, where each element (*i*,*j*) represents the mean topological dissimilarity between subjects from age groups *G*_*i*_ and *G*_*j*_.

This matrix reveals several key findings. The highest intergroup dissimilarity was observed between neonates and adolescents, suggesting that early-life HRV structure is not only quantitatively but also qualitatively distinct from later developmental stages. Intragroup variability, reflected in the diagonal elements, was maximal in neonates and early infancy, confirming greater heterogeneity in HRV topological structure during early development, possibly reflecting individual variability in maturation rates of the autonomic nervous system. A gradual reduction in both intra- and intergroup variability is observed with increasing age, particularly beyond the toddler stage, suggesting a developmental stabilization of HRV structure and convergence toward a normative topological profile. An asymmetric dissimilarity trend is also noticeable: dissimilarity between infants and school-age children is higher than between preschoolers and adolescents, implying that the most pronounced topological shift occurs during the infancy-to-childhood transition rather than adolescence.

Collectively, these results establish that topological complexity in HRV is dynamically modulated across pediatric development. Early life is characterized by a small number of strong, diverse, and heterogeneous recurrent cardiac patterns, while later stages exhibit attenuated and more homogeneous topological structures. These observations indicate the underlying maturation of autonomic control and support the utility of topological descriptors as sensitive biomarkers of developmental physiological states.

### 4.3 Topological features correlate with conventional physiological HRV indices

To assess the physiological significance of the topological features extracted from HRV signals, we computed Pearson correlation coefficients between the persistent homology descriptors and standard time-domain HRV metrics, all log-transformed to ensure approximate normality and variance homogeneity.

Specifically, we examined associations between topological variables and the natural logarithms of ${Mean}_{RR}$, ${SDNN}_{RR}$, ${RMSSD}_{RR}$, and ${PNN50}_{RR}$, and chronological age.

**Fig 6:**
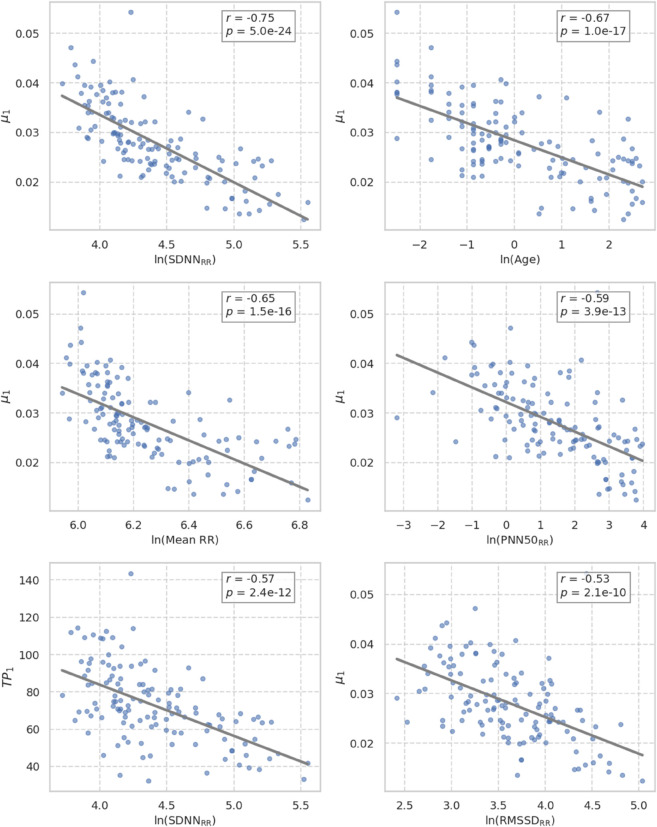
Scatter plots illustrating representative correlations between topological and log-transformed HRV features.

Fig 6 represents the six strongest statistically significant correlations (|r|>0.52), revealing a consistent and robust relationship between the geometry of HRV dynamics and conventional autonomic indices. Each subplot reports Pearson’s *r* and *p*-value (Holm-adjusted).

Notably, the mean persistence $\mu_{1}$ exhibited the highest negative correlation with both HRV and age-related indices: *r* = −0.75 with $\ln({SDNN}_{RR})$, *r* = −0.67 with ln(Age), and *r* = −0.65 with $\ln({Mean}_{RR})$ (all *p* < 10^−10^, Holm-adjusted). These findings suggest that increased HRV and younger age are associated with shorter average lifetimes of topological cycles, reflecting more rapid, transient, and less geometrically stable oscillatory dynamics, consistent with a less structured but more adaptable autonomic regulation in early development.

Other descriptors also demonstrated physiologically significant associations (see the supplementary information in S3 File for the full correlation table). Total persistence (*TP*_1_) showed moderate inverse correlations with $\ln({SDNN}_{RR})$ (*r* = −0.57), indicating that more complex and long-lived topological structures tend to occur in individuals with reduced HRV. The number of persistent features (*N*_1_) was positively associated with $\ln({PNN50}_{RR})$ (*r* = 0.49), ln(Age) (*r* = 0.41), and $\ln({SDNN}_{RR})$ (*r* = 0.40), suggesting that a greater number of topological events, albeit short-lived, co-occurs with enhanced beat-to-beat variability. Persistence entropy (*PE*_1_), representing the heterogeneity of cycle lifetimes, showed consistent positive correlations with all classical HRV measures (ranging from *r* = 0.32 to 0.43), indicating that higher signal irregularity is associated with broader spectral variability in RR intervals.

In sum, these results provide convergent evidence that topological complexity captures physiologically meaningful properties of HRV, and that persistent homology offers descriptors that are not only statistically robust but also physiologically interpretable across development.

### 4.4 Topological dissimilarity patterns across pediatric age groups

To further explore the relationship between topological variability and conventional cardiac autonomic dynamics, we evaluated the intra-group landscape dispersion. This measure, corresponding to the diagonal elements of the distance matrix in Fig 5, quantifies the average pairwise Euclidean distance between persistence landscapes within each age group, denoted as ${\bar{d}}_{G_{i},\;G_{i}}$.

**Fig 7:**
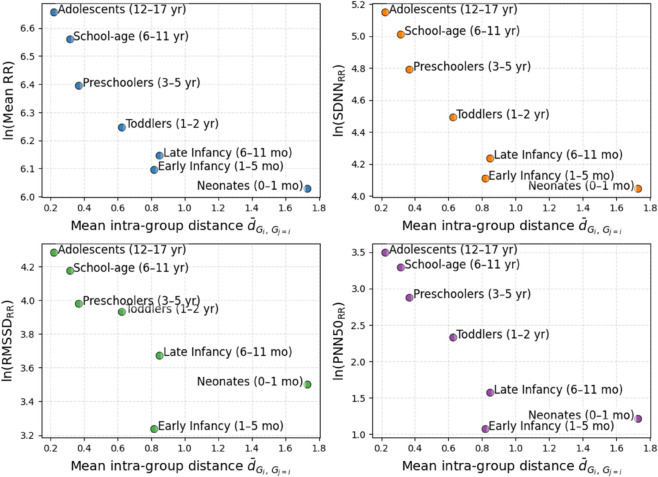
Relationship between intra-group topological variability and conventional HRV indices across developmental stages.

As illustrated in Fig 7, a clear inverse association emerged between intra-group topological variability and conventional HRV indices. Developmental stages characterized by higher internal topological heterogeneity, notably neonates and early infants, exhibited lower HRV values, including $\ln({Mean}_{RR})$, $\ln({SDNN}_{RR})$, $\ln({RMSSD}_{RR})$, and $\ln({PNN50}_{RR})$. In contrast, school-age children and adolescents showed lower intra-group topological dispersion, reflecting more homogeneous and structured cardiac dynamics, accompanied by elevated HRV levels.

This inverse relationship suggests that as the autonomic nervous system matures, the cardiac system becomes not only more effective (higher HRV) but also more geometrically regular and topologically compact. Thus, topological descriptors capture complementary information beyond mean HRV levels, reflecting the structural organization and coordination of the underlying physiological system.

Importantly, this pattern supports the hypothesis that early developmental stages are characterized by greater flexibility and variability in autonomic output, as evidenced by the broad diversity of topological profiles, whereas later stages reflect a consolidation of cardiac control mechanisms, leading to HRV signatures that are both topologically and physiologically stable.

## 5 Discussion

This study provides compelling evidence that topological features derived via persistent homology from time-delayed embedded HRV signals reflect critical developmental transitions in autonomic cardiac regulation across the pediatric lifespan. By applying topological data analysis to RR interval time series of subjects ranging from neonates to adolescents, we identified robust descriptors—such as the number of persistent cycles, mean persistence, and persistence landscapes—that capture both intra-group variability and inter-group divergence in cardiac dynamics with developmental sensitivity.

Our findings indicate that neonates and early infancy (0–5 months) exhibit the richest and most heterogeneous topological structure, characterized by a high number of persistent one-dimensional cycles (*N*_1_), elevated mean persistence ($\mu_{1}$), and substantial intra-group topological dissimilarity. These features suggest a cardiac dynamic regime dominated by prolonged, recurrent structures in the embedded phase space, possibly reflecting immature, less constrained autonomic control. Notably, the diagonal elements of the pairwise persistence landscape distance matrix confirmed this heterogeneity, indicating significant dispersion in topological features within the youngest cohorts.

In contrast, older age groups (particularly school-age children and adolescents) demonstrated a marked reduction in topological variability, both within and between individuals. This was evidenced by a lower number of persistent cycles, decreased mean and total persistence values, flatter and more homogeneous average persistence landscapes (Fig 4), and a convergence in pairwise distances (Fig 5).

These topological results are consistent with the conventional indices, which exhibited the expected developmental trends. Mean *RR* and SDNN_*RR*_ increased from infancy to adolescence, while pNN50_*RR*_ reflected greater vagal modulation and autonomic balance in older groups (Table 2). Notably, RMSSD_*RR*_ did not differ significantly between groups, suggesting a relatively earlier stabilization of short-term vagal modulation, even as longer-term variability continues to evolve. The parallel trajectories of conventional and topological measures support that the observed topological transitions reflect genuine physiological maturation rather than analytical artifacts.

These findings support the physiological interpretation that maturation of autonomic nervous system function is accompanied by a consolidation of cardiac control, resulting in more regular, efficient, and topologically compact dynamics. This trajectory is consistent with known developmental trends involving baroreflex maturation, vagal tone stabilization, and refinement of sympathetic–parasympathetic interactions.

Compared with other nonlinear HRV methods—such as entropy-based indices, fractal analyses, and recurrence quantification analysis (RQA)—our approach characterizes the global, multiscale geometry of cardiac dynamics rather than local irregularity or recurrence density [12,15,51,52]. While entropy and fractal measures summarize variability and correlation structure, they may overlook higher-order relationships that govern phase-space evolution. By applying persistent homology, we identify salient topological structures—specifically one-dimensional cycles (loop structures)—that reflect multiscale physiological coordination [34,37,46]. These descriptors are noise-robust, interpretable, and complementary to conventional HRV metrics, suggesting potential clinical utility as non-invasive markers of autonomic maturation and early dysregulation [4,6,8].

The persistence landscape framework proved instrumental in elucidating these patterns. Group-averaged landscape curves revealed high-amplitude, broad-bandwidth features in early development, indicative of strong and diverse recurrent geometries, while adolescents exhibited markedly lower and more uniform landscape magnitudes, reflecting attenuation and homogenization of cyclic structures. Furthermore, the top-left region of the distance matrix (corresponding to early groups) consistently showed the highest inter-group dissimilarity, underscoring the distinctiveness of neonatal and infant HRV dynamics within the topological feature space.

From a physiological perspective, we observed that the topological descriptors were strongly correlated with conventional HRV metrics, supporting their interpretability and biological relevance. Mean persistence ($\mu_{1}$) exhibited strong negative correlations with $\ln({SDNN}_{RR})$, $\ln({Mean}_{RR})$, and ln(Age), suggesting that as the heart rate signal becomes more regular and autonomic maturation progresses, recurrent topological features become shorter-lived. Total persistence and the number of persistent features were also negatively associated with age and positively linked to HRV variability indices, indicating that individuals with richer cardiac variability patterns exhibit greater structural diversity in their signal’s phase-space geometry. Interestingly, persistence entropy (*PE*_1_)—a measure of structural disorder—was positively associated with HRV indices, but with weaker effect sizes, suggesting a nuanced relationship between diversity and physiological adaptability.

A particularly novel observation was the inverse relationship between intra-group topological variability and conventional HRV metrics (Fig 7). Groups with greater inter-individual topological dispersion, notably neonates and infants, exhibited lower HRV values, while groups with higher HRV (e.g., school-age children and adolescents) displayed greater topological consistency. This suggests that physiological maturation leads to both increased autonomic efficiency and convergence toward a common structural template, reducing inter-individual variability in cardiac dynamics.

The nonparametric statistical tests (Table 3) further reinforce these insights. Kruskal–Wallis tests revealed significant groupwise differences for all topological descriptors (*p* < 0.005), and Dunn’s post hoc comparisons confirmed that the largest contrasts occurred between neonates and older groups, particularly adolescents. These results demonstrate that TDA descriptors are sensitive to discrete developmental transitions, offering a new lens through which to interpret physiological maturation.

From a methodological standpoint, the use of persistence landscapes and Euclidean inter-landscape distances adds both interpretability and statistical tractability to TDA in biomedical time series. Unlike raw persistence diagrams, landscapes provide smooth, continuous, and averagable representations of topological structure, while the associated distance metrics enable quantification of group-level similarities and heterogeneity, enhancing comparative analyses.

Collectively, these methodological advantages position persistent homology as a physiologically informed and computationally robust framework for HRV analysis. By quantifying the global, multiscale geometry of cardiac dynamics—features not readily captured by conventional or other nonlinear metrics—TDA provides a complementary perspective on autonomic maturation. The age-related topological compactness and decreased inter-individual heterogeneity observed here are consistent with biological systems evolving toward greater efficiency and stability, a hallmark of developmental homeostasis.

Extending this framework to fetal HRV could enable direct comparisons between term and preterm maturation patterns and provide early markers of autonomic development prior to birth. Given the higher fetal heart rates and modality-specific noise characteristics, future studies will adapt preprocessing and embedding parameters accordingly, incorporate gestational-age–stratified analyses, and evaluate whether persistence-based *H*_1_ descriptors robustly distinguish maturation status under non-parametric testing with multiple-comparison control. Such analyses would clarify the developmental sensitivity of topological summaries and their potential clinical utility in perinatal risk stratification. Such extensions could enhance our understanding of autonomic plasticity, physiological resilience, and early pathophysiological deviations in childhood, thereby broadening the utility of TDA in pediatric biomedical research.

## 6 Conclusion

This study demonstrates that topological data analysis, specifically persistent homology, provides a rigorous and physiologically grounded framework to characterize developmental transformations in cardiac autonomic regulation. By analyzing RR-interval time series across pediatric stages, we identified consistent, interpretable age-related changes in the geometry of HRV dynamics. *H*_1_ descriptors—number and persistence of cycles and persistence entropy—were sensitive to developmental stage and correlated strongly with conventional HRV indices, underscoring their physiological validity.

Phenotypically, early life was marked by higher topological complexity and between-subject heterogeneity, suggesting immature and variable autonomic regulation, whereas older children and adolescents presented more compact and homogeneous profiles, consistent with increasing regulatory efficiency and stability. These findings indicate that persistent homology yields multiscale, geometrically informed insights beyond traditional HRV analyses and may support early detection of atypical autonomic maturation as well as longitudinal pediatric monitoring.

## Supporting information

S1 File Estimation and validation of embedding parameters and Topological sensitivity analysis. (DOCX)

S2 File Post-hoc Dunn’s test results. (DOCX)

S3 File Correlations between topological descriptors of homology-1 and log-transformed physiological metrics. (DOCX)
