# Supplementary material for: Age-dependent patterns of cardiac complexity unveiled by topological data analysis of pediatric heart rate variability
Source: PLoS One. 2025 Dec 2;20(12):e0337620. doi: 10.1371/journal.pone.0337620 (PMC12671824; doi:10.1371/journal.pone.0337620)
Supplement: S1 File — (DOCX) [file pone.0337620.s001.docx]

# S1 File: Estimation and validation of embedding parameters and Topological sensitivity analysis.

## S1. Estimation and validation of embedding parameters

To reconstruct the phase-space of RR interval dynamics, the optimal embedding parameters were estimated for each developmental group using the combination of mutual information (MI) and false nearest neighbors (FNN) methods. Table S1 summarizes the median and interquartile ranges (IQR) of the time delay (τ_opt) and embedding dimension (d_opt) obtained across the pediatric sample.

| **Age Group** | **n** | **τ_opt** | **IQR** | **d_opt** | **IQR** |
| --- | --- | --- | --- | --- | --- |
| Neonates (0–1 mo) | 8 | 11 | (8–14) | 7 | (6–7) |
| Early Infancy (1–5 mo) | 33 | 11 | (9–12) | 6 | (6–7) |
| Late Infancy (6–11 mo) | 29 | 12 | (9–14) | 6 | (6–7) |
| Toddlers (1–2 yr) | 22 | 11 | (8–13) | 6 | (5–6) |
| Preschoolers (3–5 yr) | 10 | 9 | (6–11) | 6.5 | (6–7) |
| School-age (6–11 yr) | 15 | 9 | (6–11) | 6 | (5–7) |
| Adolescents (12–17 yr) | 10 | 7.5 | (5–9) | 6 | (5–6) |
| Total | 127 | -- | -- | -- | -- |

Table S1: Optimal time delay (τ_opt) and embedding dimension (d_opt) obtained across the pediatric sample.

The optimal delay values ranged between 7 and 12 beats, aligning with the first zero-crossing of the autocorrelation function in physiological HRV series. The optimal embedding dimensions (d_opt) concentrated around 6–7.

## S2. Comparison between FNN and Cao’s method

Table S2 and Fig S1 illustrate the comparison between the FNN criterion and Cao’s quantitative measures E1 and E2 (Cao, 1997). While the FNN approach identified stabilization around d* ≈ 6, Cao’s method consistently reached saturation of E1 and stability of E2 at d* ≈ 9. This slight overestimation is expected, as Cao’s criterion is more conservative in detecting complete unfolding, whereas FNN emphasizes dynamic separation.

| **Age Group** | **n** | **d*_FNN (IQR)** | **d*_Cao (IQR)** |
| --- | --- | --- | --- |
| Neonates (0–1 mo) | 8 | 7 (6–7) | 9 (8–9) |
| Early Infancy (1–5 mo) | 33 | 6 (6–7) | 9 (2–9) |
| Late Infancy (6–11 mo) | 29 | 6 (6–7) | 9 (8–9) |
| Toddlers (1–2 yr) | 22 | 6 (5–6) | 9 (8–9) |
| Preschoolers (3–5 yr) | 10 | 6 (6–9) | 9 (3–9) |
| School-age (6–11 yr) | 15 | 6 (5–9) | 9 (9–9) |
| Adolescents (12–17 yr) | 10 | 6 (5–6) | 9 (1–9) |
| Total | 127 | -- | -- |

##
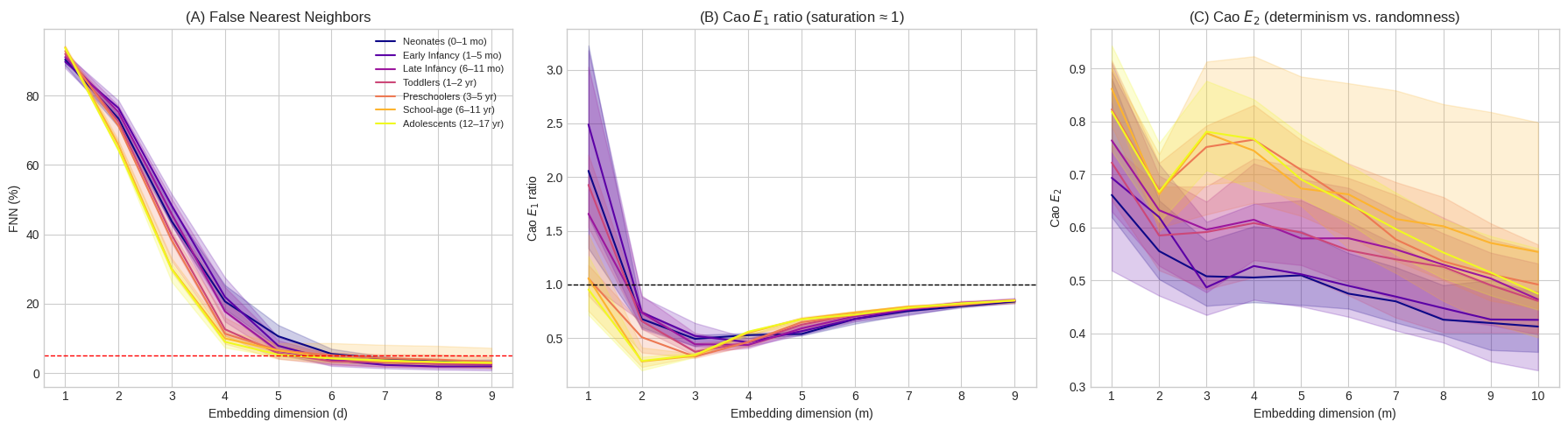


## Supplementary Figure S1. Comparison of embedding dimension estimation methods across developmental age groups. (A) False Nearest Neighbors (FNN) criterion, showing a monotonic decay and stabilization below the 5% threshold around d ≈ 6. (B) Cao’s E₁ ratio, which saturates near 1 for d ≥ 9. (C) Cao’s E₂ statistic, used to differentiate deterministic from stochastic behavior, remains below 1 for all groups. Shaded regions represent the standard deviation across subjects within each age group.

## S3. Topological sensitivity analysis

Following Maletić et al. (2016), a qualitative sensitivity analysis was conducted by computing topological descriptors across embeddings d=2–6. Persistence entropy (PE_k) increased sharply between d=2 and d=3, then reached a stable plateau for higher embeddings (see Fig S2). This stability indicates that the essential topological invariants (loops and their persistence distribution) remain consistent beyond d=3, confirming it as the minimal topologically sufficient embedding dimension- adequately capturing the intrinsic complexity of heart rate variability while avoiding redundant geometric information.


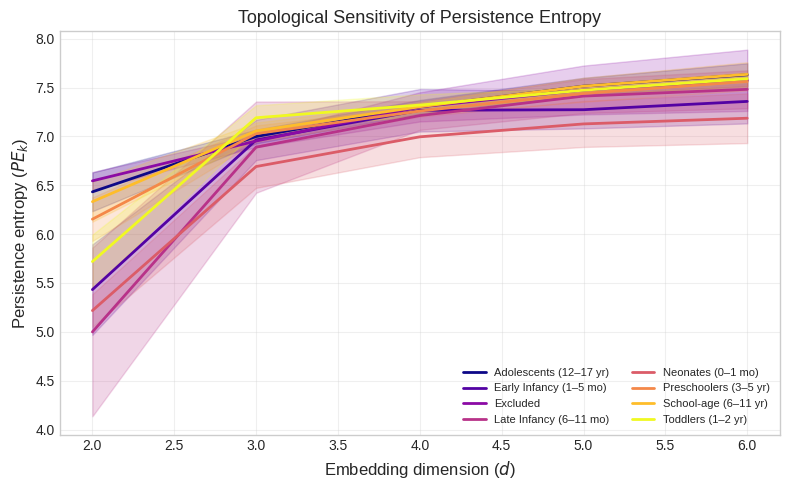


Supplementary Figure S2. Topological sensitivity of persistence entropy across embedding dimensions.

## References (Supplementary)

- Fraser, A. M., & Swinney, H. L. (1986). Independent coordinates for strange attractors from mutual information. Physical Review A, 33(2), 1134–1140. <https://doi.org/10.1103/PhysRevA.33.1134>
- Cao, L. (1997). Practical method for determining the minimum embedding dimension of a scalar time series. Physica D: Nonlinear Phenomena, 110(1–2), 43–50. <https://doi.org/10.1016/S0167-2789(97)00118-8>
- Maletić, S., Zhao, Y., Rajković, M., & Jost, J. (2016). Persistent topological features of dynamical systems. Chaos, 26(5), 053105. <https://doi.org/10.1063/1.4949472>
