# Supplementary material for: Age-dependent patterns of cardiac complexity unveiled by topological data analysis of pediatric heart rate variability
Source: PLoS One. 2025 Dec 2;20(12):e0337620. doi: 10.1371/journal.pone.0337620 (PMC12671824; doi:10.1371/journal.pone.0337620)
Supplement: S2 File — (DOCX) [file pone.0337620.s002.docx]

**S2 File: Post-hoc Dunn’s Test Results**

This file contains additional supporting information for the manuscript. Post-hoc Dunn’s Test Results.

**S1 Table. Post-hoc Dunn’s test (Holm-adjusted p-values) for N1 (Number of Persistent Elements). Kruskal–Wallis p = 0.0005**

|  | Neonates | Early Inf. | Late Inf. | Toddlers | Presch. | School-age | Adol. |
| --- | --- | --- | --- | --- | --- | --- | --- |
| Neonates (0–1 mo) | 1.0000 | 1.0000 | 0.8849 | 0.0343 | 0.0113 | 0.0668 | 0.1894 |
| Early Infancy (1–5 mo) |  | 1.0000 | 1.0000 | 0.1101 | 0.0463 | 0.2962 | 0.9044 |
| Late Infancy (6–11 mo) |  |  | 1.0000 | 0.9044 | 0.2983 | 1.0000 | 1.0000 |
| Toddlers (1–2 yr) |  |  |  | 1.0000 | 1.0000 | 1.0000 | 1.0000 |
| Preschoolers (3–5 yr) |  |  |  |  | 1.0000 | 1.0000 | 1.0000 |
| School-age (6–11 yr) |  |  |  |  |  | 1.0000 | 1.0000 |
| Adolescents (12–17 yr) |  |  |  |  |  |  | 1.0000 |

**S2 Table. Post-hoc Dunn’s test (Holm-adjusted p-values) for TP1 (Total Persistence). Kruskal–Wallis p = 0.0000**

|  | Neonates | Early Inf. | Late Inf. | Toddlers | Presch. | School-age | Adol. |
| --- | --- | --- | --- | --- | --- | --- | --- |
| Neonates (0–1 mo) | 1.0000 | 1.0000 | 1.0000 | 1.0000 | 0.2699 | 0.2636 | 0.0609 |
| Early Infancy (1–5 mo) |  | 1.0000 | 1.0000 | 0.7660 | 0.0847 | 0.0533 | 0.0075 |
| Late Infancy (6–11 mo) |  |  | 1.0000 | 1.0000 | 0.4845 | 0.4062 | 0.0723 |
| Toddlers (1–2 yr) |  |  |  | 1.0000 | 1.0000 | 1.0000 | 0.6711 |
| Preschoolers (3–5 yr) |  |  |  |  | 1.0000 | 1.0000 | 1.0000 |
| School-age (6–11 yr) |  |  |  |  |  | 1.0000 | 1.0000 |
| Adolescents (12–17 yr) |  |  |  |  |  |  | 1.0000 |

**S3 Table. Post-hoc Dunn’s test (Holm-adjusted p-values) for MP1 (Max Persistence). Kruskal–Wallis p = 0.0048**

|  | Neonates | Early Inf. | Late Inf. | Toddlers | Presch. | School-age | Adol. |
| --- | --- | --- | --- | --- | --- | --- | --- |
| Neonates (0–1 mo) | 1.0000 | 1.0000 | 0.1215 | 0.9372 | 0.1516 | 0.0158 | 0.0156 |
| Early Infancy (1–5 mo) |  | 1.0000 | 1.0000 | 1.0000 | 1.0000 | 0.3344 | 0.3199 |
| Late Infancy (6–11 mo) |  |  | 1.0000 | 1.0000 | 1.0000 | 1.0000 | 1.0000 |
| Toddlers (1–2 yr) |  |  |  | 1.0000 | 1.0000 | 0.7640 | 0.6371 |
| Preschoolers (3–5 yr) |  |  |  |  | 1.0000 | 1.0000 | 1.0000 |
| School-age (6–11 yr) |  |  |  |  |  | 1.0000 | 1.0000 |
| Adolescents (12–17 yr) |  |  |  |  |  |  | 1.0000 |

S4 Table. Post-hoc Dunn’s test (Holm-adjusted p-values) for μ1 (Mean Persistence). Kruskal–Wallis p = 0.0000

|  | Neonates | Early Inf. | Late Inf. | Toddlers | Presch. | School-age | Adol. |
| --- | --- | --- | --- | --- | --- | --- | --- |
| Neonates (0–1 mo) | 1.0000 | 0.6953 | 0.1149 | 0.0021 | 0.0003 | 0.0001 | 0.0000 |
| Early Infancy (1–5 mo) |  | 1.0000 | 1.0000 | 0.0347 | 0.0046 | 0.0016 | 0.0006 |
| Late Infancy (6–11 mo) |  |  | 1.0000 | 0.6953 | 0.0820 | 0.0570 | 0.0161 |
| Toddlers (1–2 yr) |  |  |  | 1.0000 | 1.0000 | 1.0000 | 0.8310 |
| Preschoolers (3–5 yr) |  |  |  |  | 1.0000 | 1.0000 | 1.0000 |
| School-age (6–11 yr) |  |  |  |  |  | 1.0000 | 1.0000 |
| Adolescents (12–17 yr) |  |  |  |  |  |  | 1.0000 |

**S5 Table. Post-hoc Dunn’s test (Holm-adjusted p-values) for PE1 (Persistence Entropy). Kruskal–Wallis p = 0.0008**

|  | Neonates | Early Inf. | Late Inf. | Toddlers | Presch. | School-age | Adol. |
| --- | --- | --- | --- | --- | --- | --- | --- |
| Neonates (0–1 mo) | 1.0000 | 0.6255 | 0.0972 | 0.0053 | 0.0022 | 0.0389 | 0.2842 |
| Early Infancy (1–5 mo) |  | 1.0000 | 1.0000 | 0.2481 | 0.1033 | 1.0000 | 1.0000 |
| Late Infancy (6–11 mo) |  |  | 1.0000 | 1.0000 | 0.8984 | 1.0000 | 1.0000 |
| Toddlers (1–2 yr) |  |  |  | 1.0000 | 1.0000 | 1.0000 | 1.0000 |
| Preschoolers (3–5 yr) |  |  |  |  | 1.0000 | 1.0000 | 1.0000 |
| School-age (6–11 yr) |  |  |  |  |  | 1.0000 | 1.0000 |
| Adolescents (12–17 yr) |  |  |  |  |  |  | 1.0000 |
