# Supplementary material for: Age-dependent patterns of cardiac complexity unveiled by topological data analysis of pediatric heart rate variability
Source: PLoS One. 2025 Dec 2;20(12):e0337620. doi: 10.1371/journal.pone.0337620 (PMC12671824; doi:10.1371/journal.pone.0337620)
Supplement: S3 File — (DOCX) [file pone.0337620.s003.docx]

**S3 File: Correlations between topological descriptors of homology-1 and log-transformed physiological metrics**

This file contains additional supporting information for the manuscript.

**S1 Table. Correlations between topological descriptors of homology-1 and log-transformed physiological metrics. Rows marked with × indicate non-significant correlations (p ≥ 0.05).**

| TDA Feature | Physiological Metric | r | p-value | N | Sig. |
| --- | --- | --- | --- | --- | --- |
| μ1 | ln(SDNNRR) | -0.748 | 4.99 × 10⁻²⁴ | 127 | ✓ |
| μ1 | ln(Age) | -0.667 | 1.04 × 10⁻¹⁷ | 127 | ✓ |
| μ1 | ln(Mean RR) | -0.649 | 1.48 × 10⁻¹⁶ | 127 | ✓ |
| μ1 | ln(PNN50RR) | -0.587 | 3.92 × 10⁻¹³ | 127 | ✓ |
| μ1 | ln(RMSSDRR) | -0.526 | 2.12 × 10⁻¹⁰ | 127 | ✓ |
| TP1 | ln(SDNNRR) | -0.571 | 2.43 × 10⁻¹² | 127 | ✓ |
| TP1 | ln(Mean RR) | -0.483 | 8.65 × 10⁻⁰⁹ | 127 | ✓ |
| TP1 | ln(Age) | -0.471 | 2.27 × 10⁻⁰⁸ | 127 | ✓ |
| TP1 | ln(PNN50RR) | -0.359 | 3.43 × 10⁻⁰⁵ | 127 | ✓ |
| TP1 | ln(RMSSDRR) | -0.344 | 7.35 × 10⁻⁰⁵ | 127 | ✓ |
| TP1 | ln(min RR) | 0.022 | 8.06 × 10⁻¹ | 127 | × |
| TP1 | ln(max RR) | -0.076 | 3.94 × 10⁻¹ | 127 | × |
| MP1 | ln(Mean RR) | -0.373 | 1.61 × 10⁻⁰⁵ | 127 | ✓ |
| MP1 | ln(Age) | -0.364 | 2.61 × 10⁻⁰⁵ | 127 | ✓ |
| MP1 | ln(SDNNRR) | -0.362 | 2.86 × 10⁻⁰⁵ | 127 | ✓ |
| MP1 | ln(PNN50RR) | -0.318 | 2.67 × 10⁻⁰⁴ | 127 | ✓ |
| MP1 | ln(RMSSDRR) | -0.156 | 7.95 × 10⁻² | 127 | × |
| MP1 | ln(min RR) | -0.036 | 6.92 × 10⁻¹ | 127 | × |
| MP1 | ln(max RR) | 0.008 | 9.31 × 10⁻¹ | 127 | × |
| PE1 | ln(PNN50RR) | 0.434 | 3.36 × 10⁻⁰⁷ | 127 | ✓ |
| PE1 | ln(Age) | 0.361 | 3.13 × 10⁻⁰⁵ | 127 | ✓ |
| PE1 | ln(RMSSDRR) | 0.301 | 5.85 × 10⁻⁰⁴ | 127 | ✓ |
| PE1 | ln(Mean RR) | 0.298 | 6.78 × 10⁻⁰⁴ | 127 | ✓ |
| PE1 | ln(SDNNRR) | 0.318 | 2.67 × 10⁻⁰⁴ | 127 | ✓ |
| PE1 | ln(min RR) | 0.011 | 9.04 × 10⁻¹ | 127 | × |
| PE1 | ln(max RR) | -0.069 | 4.40 × 10⁻¹ | 127 | × |
| N1 | ln(PNN50RR) | 0.489 | 5.24 × 10⁻⁰⁹ | 127 | ✓ |
| N1 | ln(Age) | 0.414 | 1.31 × 10⁻⁰⁶ | 127 | ✓ |
| N1 | ln(RMSSDRR) | 0.396 | 4.02 × 10⁻⁰⁶ | 127 | ✓ |
| N1 | ln(SDNNRR) | 0.385 | 7.91 × 10⁻⁰⁶ | 127 | ✓ |
| N1 | ln(Mean RR) | 0.365 | 2.44 × 10⁻⁰⁵ | 127 | ✓ |
| N1 | ln(min RR) | 0.031 | 7.32 × 10⁻¹ | 127 | × |
| N1 | ln(max RR) | -0.034 | 7.03 × 10⁻¹ | 127 | × |
